# Reconciling Granular Hydrogel Microstructure and Mechanics via a Temporary Thermo‐Responsive and Cell‐Invadable Matrix

**DOI:** 10.1002/smll.75043

**Published:** 2026-08-06

**Authors:** Jun Kim, Soyeon Kwon, Taimoor H. Qazi

**Affiliations:** ^1^ Weldon School of Biomedical Engineering Purdue University West Lafayette Indiana USA

**Keywords:** injectable hydrogels, microgels, microparticles, tissue engineering, wound healing

## Abstract

Granular hydrogels are biomaterials composed of densely packed microparticles forming microporous structures. Their architecture can be controlled by tuning microparticle size, shape, and packing density. However, the mechanical properties of granular hydrogels mainly depend on interparticle interactions, whereby increasing the separation distance between microparticles to improve void volume weakens overall mechanical properties and induces unjamming, severely limiting their design and applicability. This study develops Thermo‐Responsive Granular Hydrogels (TRGHs) with adjustable interparticle spacing and preserved mechanical integrity through incorporating a temporary thermo‐responsive and cell‐invadable interstitial matrix. It is shown that the interstitial space can be increased by more than 150% and the storage modulus can be increased by 4 orders of magnitude from 5 to 8900 Pa and maintained at this level by first processing at 5°C and then increasing the temperature to 37°C. Crucially, TRGHs show improved extrudability and structural integrity after 3D printing, support enhanced in vitro cell migration from embedded spheroids, and permit uninhibited in vivo cell and vessel invasion after subcutaneous injection. By addressing the trade‐off between interparticle space and mechanical properties, these advanced biomaterials broaden the design possibilities for granular hydrogels in biofabrication, in vitro disease modeling, and tissue repair.

## Graphical abstract

Thermo‐responsive granular hydrogels (TRGHs), composed of microparticles and an interstitial matrix, exhibit thermo‐responsive mechanical properties and allow cell infiltration. This system effectively addresses the conventional compromise between mechanical strength and porosity observed in granular hydrogels.

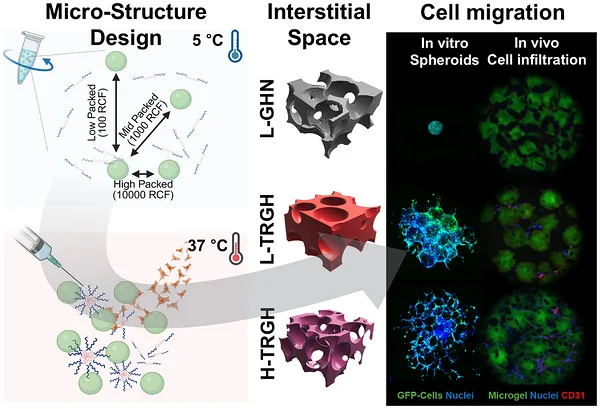

## 1 Introduction

Granular hydrogels comprising microparticle building blocks have been explored as injectable and microporous biomaterials due to their distinctive microporous architecture that facilitates cell infiltration and enhances tissue regeneration [1, 2]. The reversible interparticle interactions among microparticles make them well‐suited as injectable scaffolds for tissue engineering and extrudable inks for 3D printing applications [3]. A significant advantage of granular hydrogels is the ability to customize their porosity and microporous architecture by manipulating the size [4], shape [5], and packing density of the constituent microparticles [6]. For example, the micropore size and overall void fraction within granular hydrogels can be modulated by adjusting the microparticle diameter, and this pore size influences biological responses, including immune cell phenotype and vascularization [7, 8]. Given the critical role of porosity in granular hydrogels, previous work has shown that granular hydrogels composed of anisotropic rod‐shaped microparticles consisting of directional pores supported enhanced endothelial sprouting compared to those composed of spherical microparticles [9]. Further, engineering hierarchical porosity in granular hydrogels by incorporating porous microparticles created through polymer phase separation was found to enhance overall porosity and cell invasion [10]. Although many similar approaches have been implemented, the porosity of granular scaffolds is inversely correlated to mechanical properties such that there is a trade‐off between higher pore fractions and high mechanical strength, which can limit the potential applications of granular biomaterials.

In granular hydrogel systems, mechanical properties depend predominantly on interparticle interactions [11, 12]. Previous studies have reported that the mechanical properties of granular hydrogels can arise from physical interactions, including friction between microparticle contact surfaces [9], annealing processes [13], reversible crosslinking via dynamic hydrazone bond formation [14], and electrostatic interactions [15]. Increasing the distance between microparticles to increase hydrogel porosity can compromise these physical interactions, resulting in weakened mechanical properties of the granular hydrogel. For example, in one report, the increase in porosity from 5% to 25% by varying the degree of microparticle packing was associated with a corresponding decrease in storage modulus from ∼600 to ∼100 Pa [11]. In another example, the use of sacrificial microparticles to increase the porosity of extrusion‐printed granular inks from ∼20% to ∼60% resulted in a drastic reduction of storage modulus by an order of magnitude [16]. Furthermore, extrusion of high porosity granular hydrogel formulations through syringe needles or printing nozzles can cause the expulsion of excess interstitial fluid. In this regard, Xin et al. reported that low‐packed microparticles can undergo a shear‐induced jamming transition during extrusion, leading to pore collapse and initial water expulsion from the granular hydrogel [17]. Collectively, these are other similar studies demonstrate that achieving an optimal balance between granular hydrogel porosity and mechanical properties has been a persistent challenge in the field, creating a bottleneck for their advanced applications as biomaterial inks and tissue engineering scaffolds [18].

This issue of reconciling high porosity and mechanical strength can be partially addressed through post‐crosslinking neighboring microparticles after packing [19]; although this can often limit injectability and post‐processing [12]. One approach involves light‐induced covalent crosslinking of functional groups on the surfaces of neighboring microparticles to mechanically stabilize granular hydrogel structures [19, 20]. In addition, microporous annealed particle (MAP) gels have been developed through enzyme‐mediated annealing, which forms non‐canonical amide linkages between peptides functionalized on microparticles [13]. An alternative approach that bypasses the creation of permanent covalent bonds between microparticles involves incorporating an interstitial matrix into granular hydrogels. For example, an interstitial matrix composed of N‐acryloyl glycinamide and Carbomer 940 was incorporated into a polyethylene glycol diacrylate (PEGDA)–N‐acryloyl glycinamide (NAGA)‐based granular hydrogel to enhance mechanical properties, including viscosity and modulus, while maintaining extrudability [21]. Additionally, an RGD‐functionalized and biodegradable norbornene‐modified hyaluronic acid (NorHA) interstitial matrix was incorporated into a non‐degradable granular hydrogel to support lymphatic sprouting [22]. However, both systems require an additional photocuring step for interstitial hydrogel crosslinking. In another approach, guest‐host crosslinked interstitial matrices were designed and integrated into granular hydrogels, resulting in improved failure properties under compression testing compared to covalently crosslinked matrices due to the reversible nature of guest‐host crosslinking [23]. However, guest‐host crosslinking increases the extrusion forces required for granular hydrogels, which adversely affects cell viability when cells are encapsulated within the hydrogels [24], thereby diminishing one of the primary advantages of the granular hydrogel system. Importantly, although the incorporation of an interstitial matrix can improve the mechanical stability of granular hydrogels, in the absence of specific strategies such as dynamic reversible crosslinking and degradability, it may impede cell infiltration due to the nanoporous structure of the matrix hydrogel, potentially hindering endogenous tissue regeneration. Thus, a critical unresolved challenge in the field is ensuring that the interstitial matrix enhances mechanical support without adversely affecting cell viability and infiltration.

In this study, to broaden the design parameters of granular hydrogel systems, we describe a thermo‐responsive granular hydrogel (TRGH) platform, composed of a temporary and cell‐invadable matrix phase introduced between microparticles with modifiable interparticle distances. Among various thermo‐responsive hydrogels, including poly(ethylene glycol)‐poly(ε‐caprolactone)‐poly(ethylene glycol) (PEG‐PCL‐PEG, PECE) [25], poly(organophosphazene) [26], and poly(N‐isopropylacrylamide) [27], Pluronic F127 hydrogel was selected as the matrix due to its rapid gelation behavior, ease of dissolution, injectability, and biocompatibility [28]. We hypothesized that the thermo‐responsive interstitial matrix within the granular hydrogel will offer temporal mechanical support across a range of microparticle packing densities while allowing cellular migration and proliferation in the interstitial space. Specifically, once injected, the thermo‐responsive and reversibly crosslinked matrix hydrogel recovers its mechanical properties and provides mechanical support for the microgeometry of TRGHs. The amphiphilic molecular structure and the cell‐remodelable, physically crosslinked network of the matrix facilitate its dissolution under physiological conditions, thereby not impeding cell migration within the TRGHs. The packing density and corresponding mechanical properties of TRGHs were systematically characterized using high‐resolution confocal microscopy and oscillatory shear rheology. Furthermore, in vitro cell spheroid spreading and in vivo cell infiltration were studied to examine the impact of the interstitial matrix on cellular behavior. Collectively, these TRGHs offer a versatile and promising platform to address the design limitations of granular biomaterials by overcoming the trade‐off between mechanical properties and porosity.

## 2 Results and Discussion

### 2.1 Fabrication and Assembly of TRGHs

Norbornene‐modified hyaluronic acid (NorHA) was synthesized as previously described [29], and its modification was confirmed by proton nuclear magnetic resonance (^1^H‐NMR), which indicated a degree of modification of ∼32% (Figure S1). Monodisperse, spherical, and chemically crosslinked NorHA microparticles were fabricated using a flow‐focusing microfluidic device (Figure 1a). Subsequently, the fabricated microparticles were dispersed in a Pluronic F127 hydrogel solution at 5°C to inhibit physical crosslinking through micellization of the matrix hydrogel (Figure 1b). The Pluronic F127 hydrogel is a widely used biocompatible thermo‐responsive material characterized by an amphiphilic molecular structure. It exhibits temperature‐dependent micellization behavior [30] and reversible gelation properties at its lower critical solution temperature (LCST), leading to extensive applications including drug delivery, bioprinting, and cell delivery [31]. In addition, the incorporation of hyaluronic acid (HA) into Pluronic F127 micelles affects the size, packing arrangement, and structural stability of the micelles by interacting with the hydrophilic corona of the micelles [32, 33]. The amphiphilic molecular structure of the matrix hydrogel facilitates interactions with HA during stirring at 5°C [28], resulting in a dispersion without repulsive interactions between microparticles and matrix hydrogel. By adjusting the centrifugation speed between 100 and 10 000 relative centrifugal force (RCF) during the packing procedure at 5°C, TRGHs can be formed with controlled interparticle spacing (Figure 1c,d). When the temperature increases to 37°C, the hydrophobic regions of the matrix hydrogel aggregate and transition to a gel state while maintaining the microparticle packing density (Figure 1e). Furthermore, due to the reversible physical crosslinking via micellization of the matrix hydrogel, these TRGH constructs can be extruded even at 37°C, and by filling the interparticle spaces, the microstructure of the TRGHs can be preserved (Figure 1f).

**FIGURE 1:**
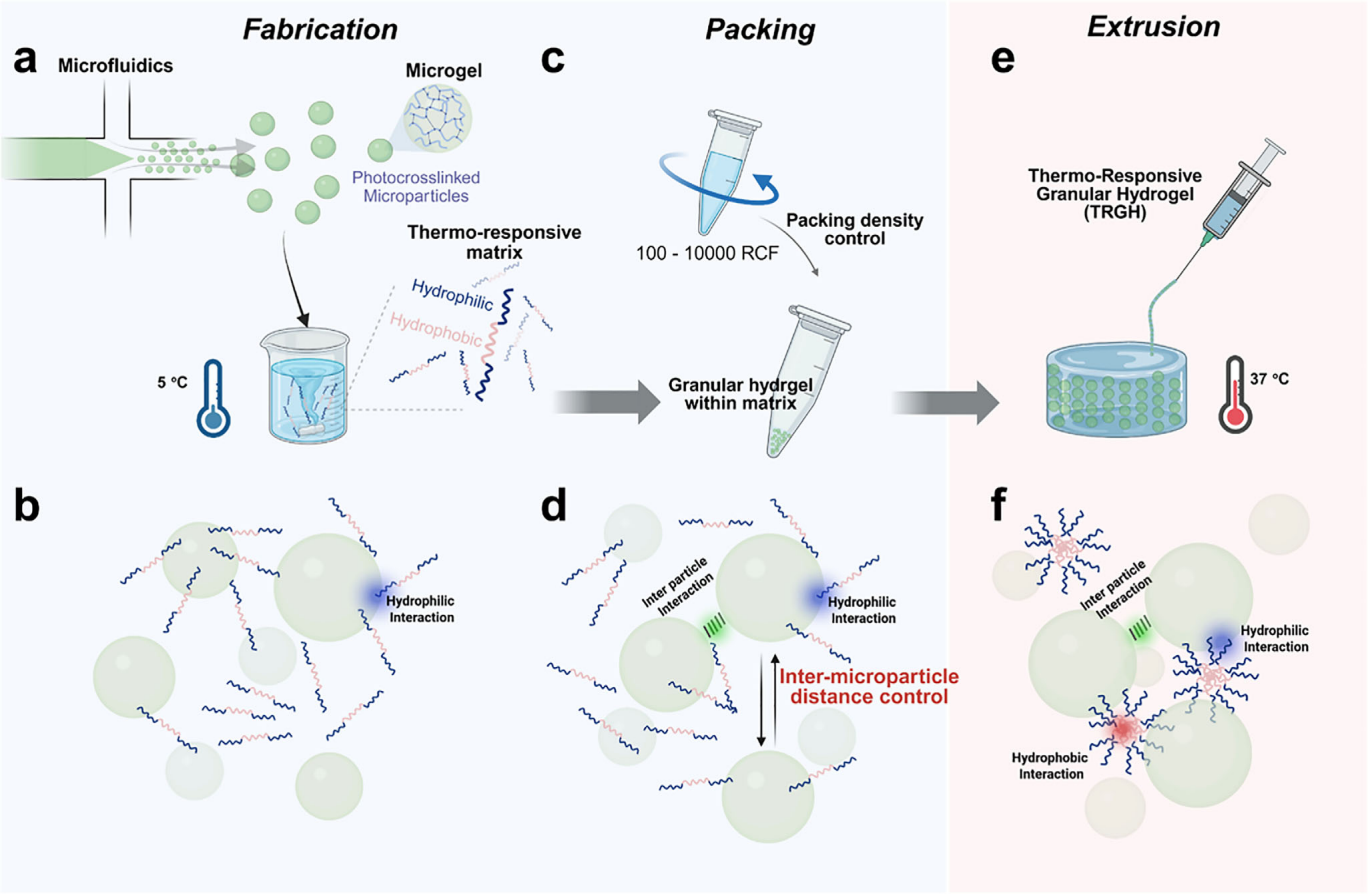
Overview of thermo‐responsive granular hydrogels (TRGHs) fabrication and their molecular interactions. (a) Fabrication of monodisperse microparticles using a microfluidic technique, followed by their incorporation into the liquid phase of a thermo‐responsive matrix precursor solution maintained at 5°C. (b) Hydrophilic interactions occur between the microparticles and the amphiphilic polymer chains of the interstitial phase. (c) Regulation of microparticle packing density through adjustment of centrifugation speed at 5°C. (d) Physical interactions among microparticles in conjunction with hydrophilic interactions between the microparticles and the interstitial phase. (e) Formation and extrusion of TRGHs at 37°C. (f) Thermo‐responsive crosslinking via micellization of the matrix hydrogel facilitated by hydrophobic interactions along with interparticle interactions, and hydrophilic interactions between the microparticles and the matrix hydrogel.

### 2.2 Modulation of Interparticle Spacing in TRGHs

To characterize the interparticle spacing of TRGHs with varying packing densities, microparticles were labeled with a fluorescent Cy3 tag, mixed with the Pluronic phase as described above, and packed at different centrifugation speeds to create granular hydrogels. Subsequently, 3D volumetric z‐stacks were acquired with confocal microscopy to characterize the microparticle morphology and interparticle spacing as in previous reports [11, 34] (Figure 2). Confocal slices confirmed monodisperse microparticles produced via microfluidics and an increase in microparticle density corresponding to higher RCF values (Figure 2a). 3D rendered images of the interparticle space further revealed a reduction in spacing with increasing packing density in TRGH groups (Figure 2b, Movie S1). To investigate a well‐known issue in this field concerning the reduction of porosity during the extrusion process, the interparticle spaces were quantified and compared before and after extrusion (Figure 2c, d). This issue was most notable in granular hydrogels with a low degree of microparticle packing; these hydrogels were packed at 100 RCF (GH_100) and exhibited a significant reduction in interparticle spacing after extrusion through a 27G needle, decreasing from ∼42.5% to ∼34.4% after extrusion. This phenomenon can be attributed to the shear stress experienced by the granular hydrogels during extrusion, which initially expels the interstitial solvent, followed by the compaction of microparticles into a denser arrangement [17]. The inclusion of the Pluronic phase solved this problem, and there were no significant differences in the interstitial spaces of TRGH groups before and after extrusion, suggesting that the interstitial matrix between microparticles stabilizes the spacing even under shear stress induced by extrusion. By modifying centrifugation speed, we obtained TRGHs with post‐extrusion interparticle spaces in the range of ∼20%–50% (Figure 2d). Due to the significant differences observed in the interparticle spacing among TRGH_100, TRGH_1000, and TRGH_10000 (Figure S2), these samples were classified as low‐ (∼47% interparticle space), mid‐ (∼32% interparticle space), and high‐(∼22% interparticle space) packed TRGH (L‐TRGH, M‐TRGH, and H‐TRGH, respectively) for subsequent experiments.

**FIGURE 2:**
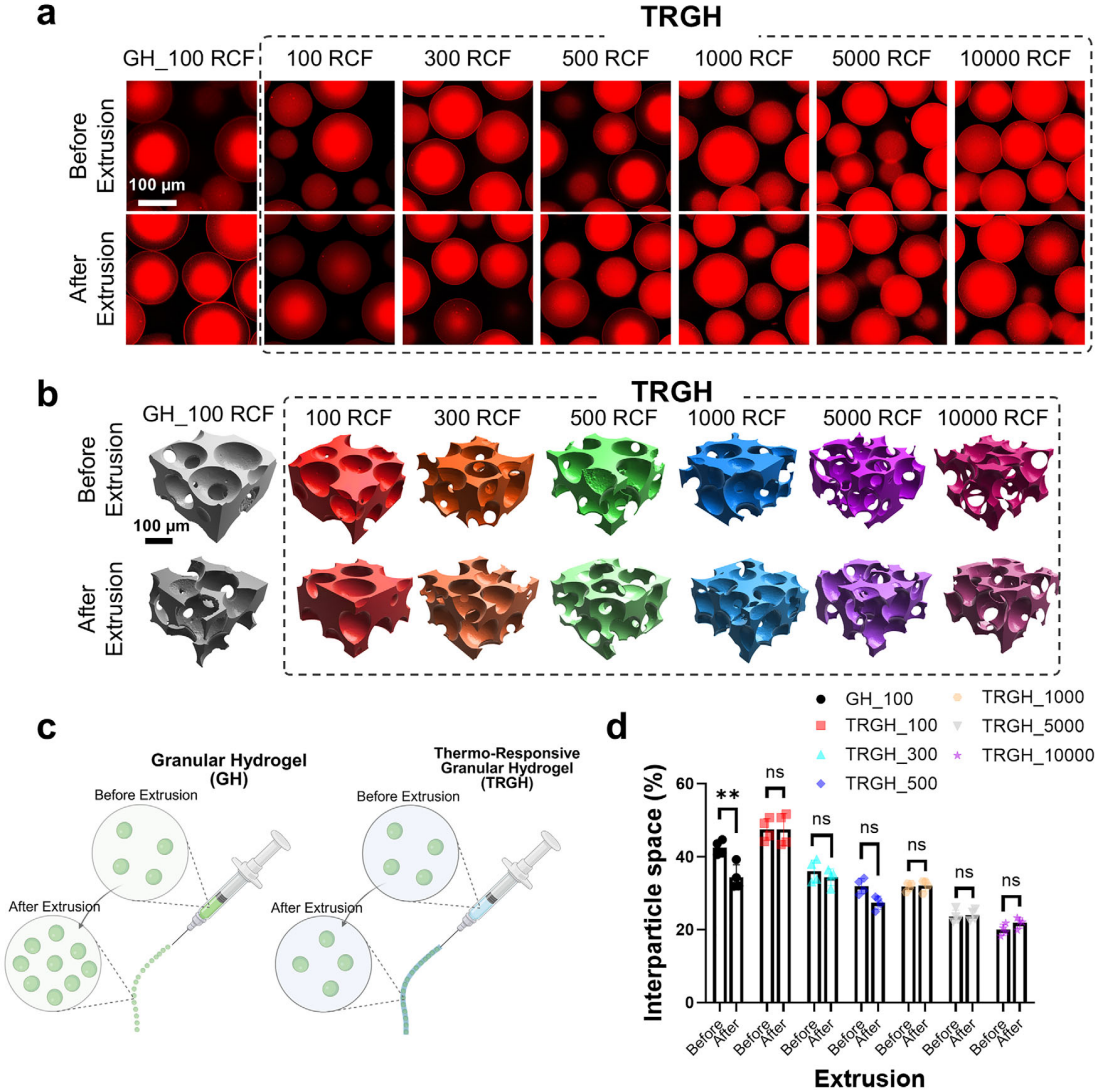
Interparticle spacing as a function of centrifugation speed in TRGHs. (a) 2D images of microparticles within GH and TRGH subjected to varying centrifugation speeds both before and after extrusion from a syringe. (b) 3D rendered images illustrating interparticle spaces within GH and TRGH. (c) Schematic illustration of changes in microstructure and interparticle space before and after extrusion of GH and TRGH. (d) Quantification of interparticle spacing in GH and TRGH before and after extrusion through a 27 G needle. ^**^ *p* < 0.01 (Analyzed by two‐way ANOVA with post hoc Tukey's multiple comparison test).

To characterize other features of the interparticle spaces within the TRGHs, the number, size, and aspect ratio of interstitial regions in GH_100 RCF and TRGH samples were analyzed both before and after extrusion (Figure S3). The average number of discrete interstitial areas within the region of interest in 2D images was recorded as ∼17.75, ∼6.25, ∼17.5, and ∼33.25 for GH_100, L‐TRGH (100 RCF), M‐TRGH (1000 RCF), and H‐TRGH (10000 RCF), respectively (Figure S3a). The minimum Feret diameter was measured at ∼50.28, ∼319.45, ∼49.14, and ∼35.67 µm for GH_100, L‐TRGH, M‐TRGH, and H‐TRGH, respectively, suggesting that large interstitial areas were connected by thin necks in highly packed granular hydrogels. Following extrusion, the number of interstitial areas, Feret diameter, minimum Feret diameter, and aspect ratios exhibited trends similar to those observed prior to extrusion in the TRGH groups (Figure S3b). To summarize, these data indicate that while conventional granular hydrogels undergo particle jamming and a reduction in porosity during syringe extrusion, the incorporation of a thermoresponsive matrix hydrogel in TRGHs stabilizes against these unwanted changes.

### 2.3 Thermo‐Responsive and Shear‐Thinning Mechanical Properties of TRGHs

Granular biomaterials are known for their shear‐responsive behaviors that permit extrusion and self‐healing. The incorporation of a distinct Pluronic phase results in granular biomaterials that are responsive to both shear and temperature, necessitating their in‐depth characterization. Pluronic has previously been used as a viscosity enhancer to aid in extrusion bioprinting [35] and it is used here as an interstitial matrix within the TRGHs, responsible for maintaining the spacing between microparticles and imparting thermo‐responsive mechanical properties, characterized by shear‐thinning behavior. To validate this, we assessed mechanical properties—including storage modulus (G’), loss modulus (G′), shear‐thinning behavior (strain sweep from 0.5% to 500% at 1 Hz), and self‐healing capacity (change in G′ and G″ after cycling through low and high strains) —using a temperature‐controlled rheometer. (Figure 3a). The granular hydrogel packed at 10000 RCF (H‐GH) exhibited a nearly constant average storage modulus of ∼421.4 Pa across a temperature range of 5°C–37°C (Figure 3b). In contrast, the Pluronic matrix remained in a sol state at low temperatures (5°C–24°C) and began to gel at approximately 24°C, reaching a storage modulus of ∼9816.6 Pa at 37°C, indicative of its thermo‐responsive mechanical behavior. Next, the rheological behavior of three TRGH groups (low, medium, and high packing) were characterized. The L‐TRGH group displayed a sol. state (G″ > G′) at low temperatures due to low packing density; however, at around 20°C, the Pluronic matrix within the L‐TRGH started to gel, with the storage modulus increasing 5 orders of magnitude to ∼8940 Pa at 37°C. The storage modulus of M‐TRGH and H‐TRGH were measured as ∼229.8 and ∼316.9 Pa at 5°C, and ∼6664.5 and ∼6293.7 Pa at 37°C, respectively. Notably, H‐TRGH demonstrated superior mechanical properties at low temperatures (5°C–20°C) compared to L‐TRGH; however, after the matrix hydrogel gelled, L‐TRGH exhibited greater mechanical properties than H‐TRGH. This indicates that at low temperatures (5°C–20°C), the mechanical properties of TRGHs are primarily determined by those of the microparticles and influenced by their packing density. However, above 20°C, these mechanical properties increase significantly due to the gelation of the Pluronic matrix. L‐TRGH exhibited significantly greater interstitial spacing and thus a higher content of matrix hydrogel compared to M‐TRGH and H‐TRGH, resulting in superior mechanical properties at 37°C.

**FIGURE 3:**
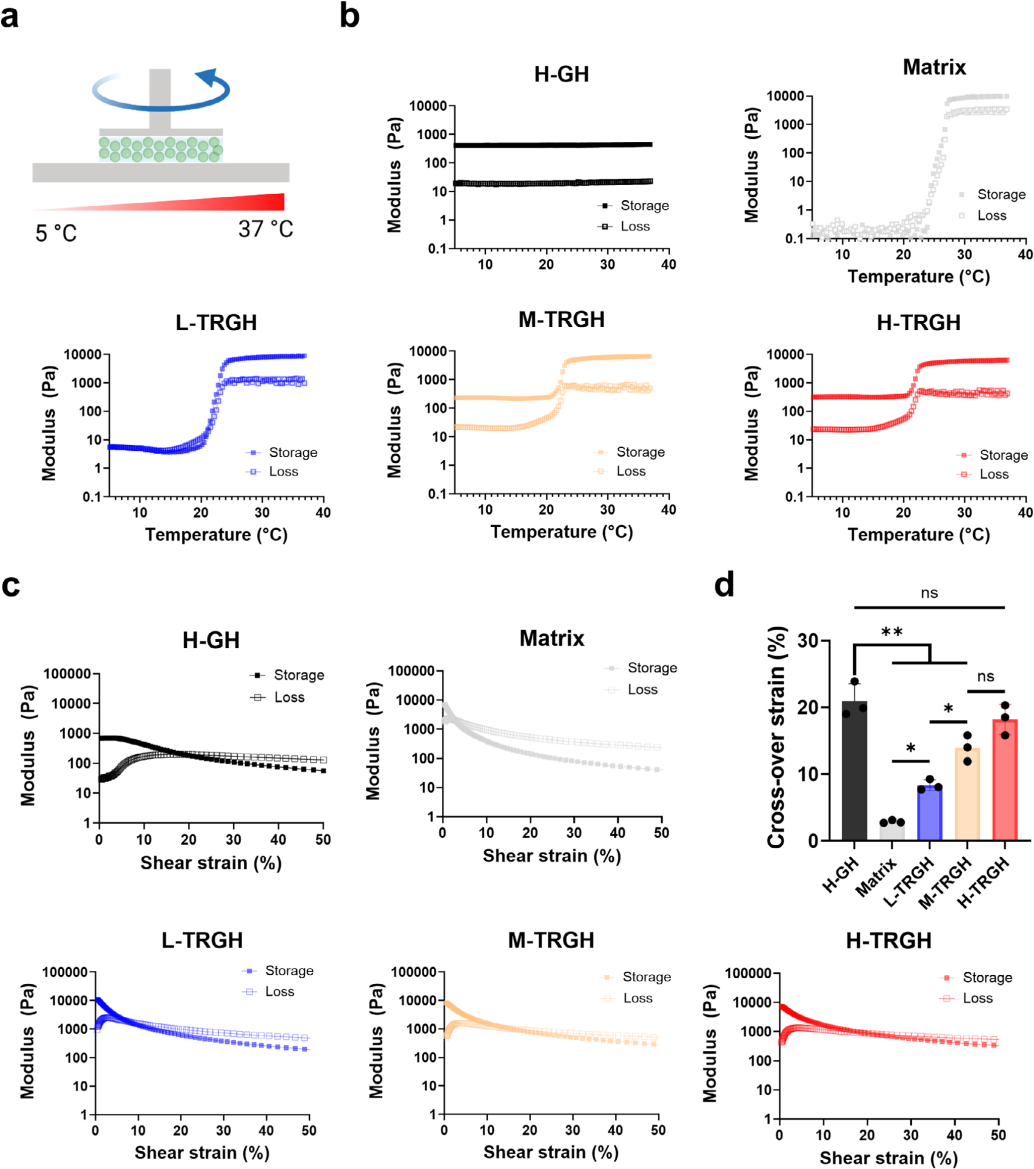
Thermo‐responsive and shear‐thinning mechanical properties of TRGHs. (a) Schematic illustration of mechanical property testing under varying temperature conditions using oscillatory shear rheology. (b) Storage and loss moduli of hydrogels measured across a temperature range from 5°C to 37°C. (c) Strain sweep test of the hydrogels, demonstrating shear‐yielding behavior at 37°C. (d) Cross‐over strain of the hydrogels determined from the strain sweep test. ^*^ *p* < 0.05, ^**^ *p* < 0.01 (Analyzed by one‐way ANOVA with post hoc Tukey's multiple comparison test).

Next, a shear strain sweep test was conducted at 37°C to examine the shear‐yielding behavior of TRGHs (Figure 3c). The modulus of H‐GH, the matrix hydrogel, and the TRGH groups decreased with increasing shear strain, and the hydrogels transitioned to dominant viscous regimes at high shear strain, demonstrating their shear‐yielding properties. Notably, the yield point, defined here as the G′ to G″ crossover strain, of the matrix hydrogel (∼2.9%) was significantly lower than that of GH (∼20.9%), (Figure 3d). The crossover strains of L‐TRGH, M‐TRGH, and H‐TRGH were ∼8.3%, ∼13.9%, and ∼18.2%, respectively, values that lie between those of GH and the matrix hydrogel. These findings suggest that the matrix hydrogel decreases the yield strain and improves injectability and shear‐thinning behavior in TRGHs compared to H‐GH. Furthermore, it is likely that, owing to stable molecular interactions between the microparticles and the amphiphilic molecular structure of the matrix hydrogel, the TRGHs exhibit mechanical properties that are homogeneous and intermediate between those of H‐GH and the matrix hydrogel. The hydrogels exhibited self‐healing properties in addition to shear‐thinning behavior, as demonstrated by the application of alternating low and high shear strains while monitoring the storage and loss modulus (Figure S4). All hydrogels transitioned to a dominant viscous regime (i.e., liquid‐like) under high strain conditions and promptly recovered to a dominant elastic regime (i.e., solid‐like) upon returning to low strain conditions. Notably, the granular hydrogel groups regained over 95% of their original mechanical properties within 2 s, whereas the matrix hydrogel group required approximately 39 s to achieve a 95% recovery (Figure S4f). Furthermore, the storage modulus of the H‐GH, L‐, M‐, and H‐TRGH groups recovered to approximately 126.9%, 116.3%, 112.2%, and 111.2%, respectively, while the matrix group exhibited a recovery of about 97.9% (Figure S4g). We hypothesize that upon the application of shear stress, the microparticles within the granular hydrogel groups undergo rearrangement, which may contribute to the slight enhancement in mechanical properties observed. These mechanical recovery and self‐healing properties are essential in the design of injectable hydrogels because, during the extrusion process, hydrogels undergo disassembly due to shear stress; however, their mechanical properties must be restored to maintain the intended 3D microenvironment [36, 37].

### 2.4 Extrusion Printing and 3D Structural Integrity of TRGHs

Granular biomaterials have found widespread applications in 3D printing and biofabrication [38]. For example, they have been used as slurries for embedded printing and as biomaterial inks for extrusion printing [3]. We initially assessed the extrudability and extrusion force prior to evaluating the 3D printability of the materials (Figure S5). The extrusion forces increased, reached a linear region, and then spiked as the syringe plunger approached the end of the barrel. Notably, due to the thermo‐responsive nature of the matrix, the TRGH groups exhibited different extrusion forces. In contrast, the L‐GH group experienced frequent clogging of the syringe needle during extrusion, likely caused by the heterogeneous settling and distribution of microparticles within the L‐GH (Figure S5a). The mean extrusion forces of L‐TRGHs were approximately 0.13 N at 5°C and 0.81 N at 37°C. Similarly, the mean extrusion forces of H‐TRGHs were 0.24 N at 5°C and 1.15 N at 37°C (Figure S5b), indicating that a higher extrusion force is required due to temperature induced physical crosslinking of the interstitial matrix. To demonstrate the potential benefits of TRGHs in 3D printing‐assisted biomaterial fabrication, we evaluated the extrudability and structural integrity of TRGHs in a temperature‐controlled extrusion‐based 3D printing setup (Figure 4a). The printing bed temperature was maintained at 37°C, while the nozzle was kept at room temperature to initiate the sol‐to‐gel transition of the matrix hydrogels within the TRGHs. Extrusion tests were performed at room temperature to assess the shape of the extruded filaments (Figure 4b). Both low packing and high packing conditions, with and without the interstitial matrix, were tested. Without the matrix, the L‐GH group extruded as droplets, resulting in poor filament formation due to insufficient microparticle packing. In contrast, the L‐TRGH group exhibited smooth filament extrusion with uniform filament thickness and high shape fidelity despite having the same packing density. The H‐GH group also produced filament extrusion; however, the filament shape was irregular. Previous work has shown that this irregularity could be caused by a number of factors including heterogenous microparticle size, packing density, wall stresses, and nozzle diameter etc., indicating that many variables must typically be optimized to obtain high fidelity printed structures with packed granular inks [39]. In comparison, the H‐TRGH group demonstrated fine filament extrusion with high regularity, resolution, and shape fidelity. The filament widths were measured to be 581.8 ± 149.7, 644.4 ± 38.3, 280.6 ± 63.5, and 548.8 ± 19.3 µm for L‐GH, L‐TRGH, H‐GH, and H‐TRGH, respectively (Figure 4c). The L‐GH exhibited dispersion, which is attributed to its limited mechanical properties, resulting in irregular and highly variable filament widths. In contrast, the H‐GH displayed narrower widths with an irregular distribution. Notably, both TRGH samples demonstrated homogeneous filament widths, with similar measurements observed between L‐TRGH and H‐TRGH. These findings indicate that regularity of filament width is strongly influenced by the packing density of the granular hydrogels, and the presence of the interstitial matrix improves the consistency and homogeneity of filament shape and width across different microparticle packing densities.

**FIGURE 4:**
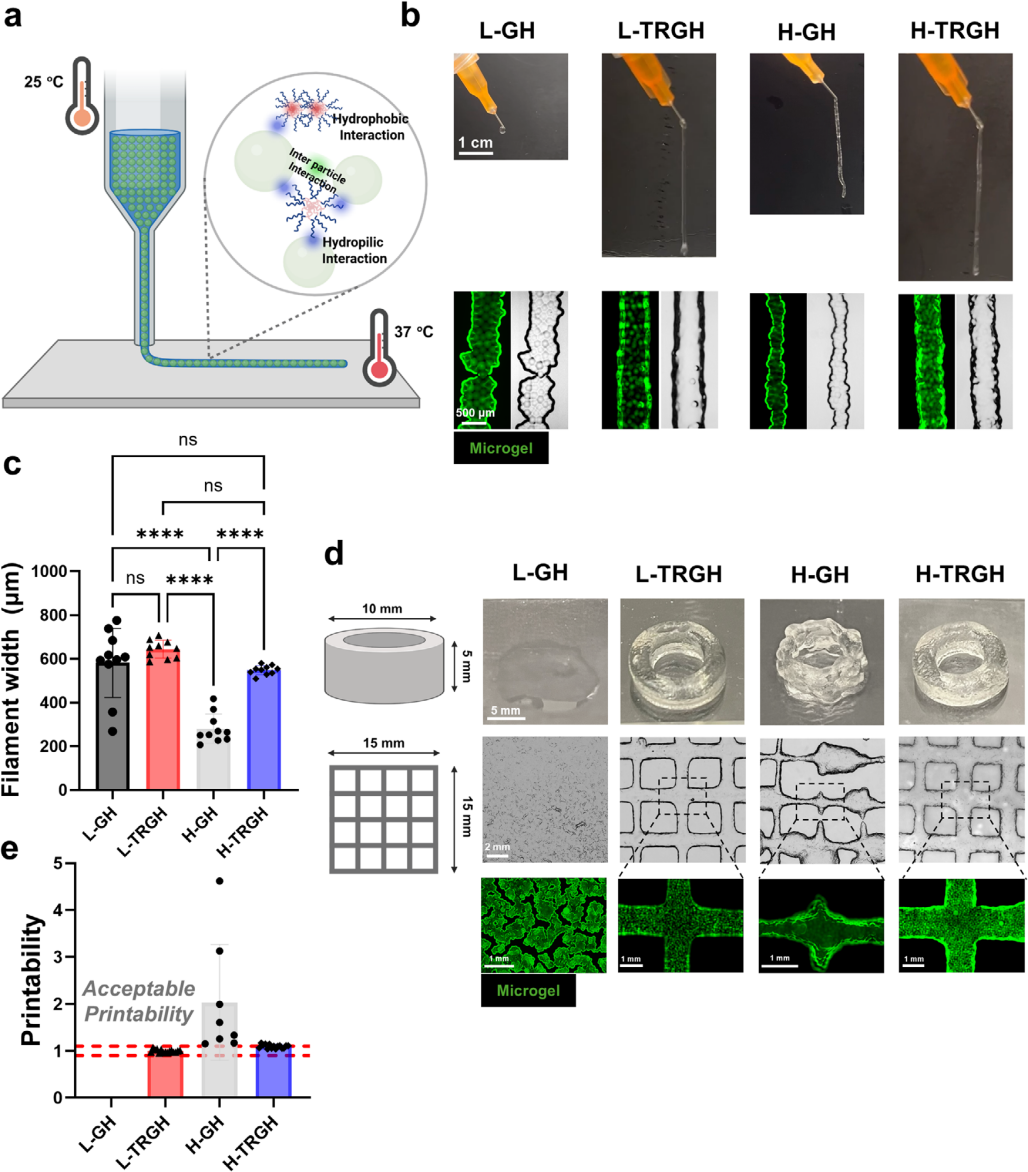
Extrusion printing and structural integrity of GHs and TRGHs. (a) Schematic illustration of a temperature‐controlled 3D printing setup and the process of printing TRGHs. (b) Representative images of syringe extrusion tests for L‐GH, L‐TRGH, H‐GH, and H‐TRGH samples conducted using a 23 G needle, along with micrographs of the resulting 3D printed filaments. (c) Quantitative assessment of 3D printed filament width. (d) Representative images and micrographs of 3D printed tubular constructs and monolayer grid structures. (e) Quantitative assessment of 3D printability based on the grid structure. ^****^ *p* < 0.0001 (Analyzed by one‐way ANOVA with post hoc Tukey's multiple comparison test).

To further validate the 3D printability and structural integrity of TRGHs, various 3D structures, including cylinders and grid patterns, were extrusion printed (Figure 4d). When printing a 5 mm‐high tubular structure, the L‐GH group failed to stack onto underlying layers due to inadequate mechanical properties to support subsequent layers, leading to structural collapse. Other groups successfully produced stacked 3D structures; however, the H‐GH group exhibited irregular surface morphology and suboptimal printability, whereas the TRGH groups showed relatively improved printability. Additionally, as anticipated, the L‐GH group failed to print grid structures, and the H‐GH group demonstrated poor print quality, while the TRGH groups successfully printed high‐quality grid structures. Printability was quantified based on the circularity of the 3D‐printed grids, following a previously reported method, where values between 0.9 and 1.1 indicate acceptable printability [40]. The printability values were ∼1.00, ∼2.03, and ∼1.10 for L‐TRGH, H‐GH, and H‐TRGH, respectively, indicating that only the TRGH groups fell within the acceptable printability range (Figure 4e). The matrix hydrogel alone exhibited good extrusion, fine filament formation, and favorable 3D printability, with a measured value of 1.00 ± 0.07 (Figure S6). Because the temperature was controlled solely through the printing bed at 37°C, samples with a higher content of interstitial matrix exhibited reduced stacking during printing. Consequently, the matrix hydrogel demonstrated the lowest stacking, whereas H‐TRGH showed the highest stacking in 3D printing (Figure S7). Because we only wanted to demonstrate the potential benefits of the TRGHs on 3D printing and not focus solely on it, we did not perform additional optimization of these experiments. However, it is possible to further improve the structural stability and print fidelity of TRGH constructs, for instance by proper regulation of the temperature in the printer space and through evaluating difference nozzle widths and morphologies. Collectively, our results suggest that the matrix hydrogel within the TRGHs enhances not only extrudability but also printing resolution and structural integrity by providing thermo‐responsive and temporary mechanical support to the hydrogels.

### 2.5 In Vitro Cells Spreading Within TRGH Scaffolds

The temporary matrix within TRGHs is expected to maintain the microarchitecture of granular hydrogels during extrusion and injection and undergo post‐injection disassembly to permit cell migration within the granular hydrogel. Prior to assessing cell spreading, cell viability was evaluated by encapsulating fibroblast spheroids and culturing them for one day within L‐GHN (a low‐packed granular hydrogel with a non‐degradable crosslinked NorHA matrix), H‐GH, L‐TRGH, and H‐TRGH. Viability measurements were approximately 78.34%, 97.38%, 93.53%, and 80.58%, respectively (Figure S8). No significant differences were observed among the H‐GH and TRGHs; however, the L‐GHN group exhibited lower viability, which may be attributed to the non‐degradable matrix limiting live cell spreading. To evaluate cell behavior within the TRGHs, spheroids assembled from GFP‐expressing NIH 3T3 fibroblasts were embedded in the hydrogels, and outward cell migration toward neighboring microparticles and into the interstitial space was characterized after three days (Figure 5a). According to previous studies, when cells are embedded within granular hydrogels, they spread throughout the interstitial spaces and undergo self‐assembly mediated by cell‐cell and cell‐material interactions [41, 42]. We hypothesized that if the matrix within the granular hydrogels constitutes a degradable and cell‐invadable network, the spheroids would spread and adhere to surrounding microparticles rather than maintaining their original spheroid morphology. To test this hypothesis, a low‐packed granular hydrogel with a non‐degradable crosslinked NorHA matrix (L‐GHN) was used as a control. All microparticles within the hydrogels were conjugated with RGD peptides to provide cell adhesion sites. The L‐GHN group exhibited spheroids that retained a round morphology and no cell migration was observed, whereas the H‐GH and TRGH groups demonstrated cell spreading and migration into the surrounding microparticles (Figure 5b). Quantitative analysis of DAPI area revealed measurements of ∼14,440.3, ∼54,667.4, ∼98,624.6, and ∼114,684.0 µm^2^ for L‐GHN, H‐GH, L‐TRGH, and H‐TRGH, respectively (Figure 5c). Similarly, GFP‐positive area quantification followed the same trend, with values of ∼12,883.7, ∼34,454.5, ∼58,881.1, and ∼70,939.5 µm^2^ for L‐GHN, H‐GH, L‐TRGH, and H‐TRGH, respectively (Figure 5d). Notably, compared to H‐GH, both L‐TRGH and H‐TRGH exhibited increased DAPI and GFP coverage areas, indicating that the matrix hydrogels within TRGHs did not adversely affect cell migration; rather, they promoted it. This phenomenon may be attributed to the mechanical stability among microparticles of TRGHs, which facilitated cell migration. Furthermore, according to previous reports, the packing density of the granular hydrogel provides varying types of void spaces to the cells, such as triangular or closed pores as well as connected necks and tunnels, thereby influencing cellular morphology [43]. In the L‐TRGH group, cells exhibited a spread morphology, whereas in the H‐TRGH group, cells displayed a highly deformed, narrow, and elongated morphology due to the confined microenvironment. These findings indicate that the TRGH system could be modulated to provide variable interstitial microenvironments that affect cellular morphology and invasion.

**FIGURE 5:**
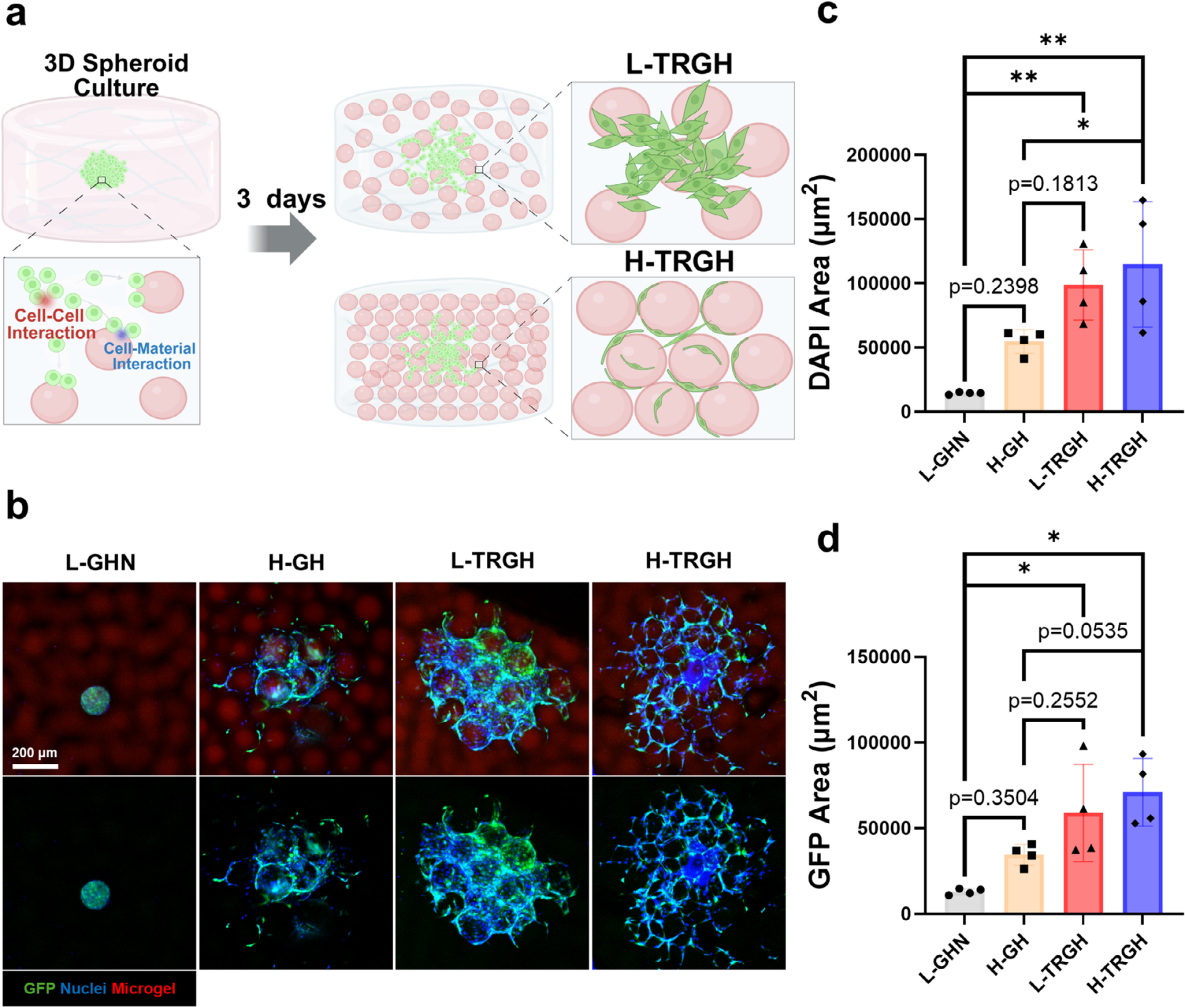
Embedding of cell spheroids and 3D culture within hydrogels. (a) Schematic illustration depicting the culture of GFP‐expressing fibroblast spheroids, highlighting cell migration and adhesion to RGD‐modified microparticles within TRGHs. (b) Representative images of spheroids cultured for three days within L‐GHN, H‐GH, L‐TRGH, and H‐TRGH hydrogels. Quantification of (c) DAPI coverage area, and (d) GFP coverage area derived from confocal imaging of the spheroids. ^*^ *p* < 0.05, ^**^ *p* < 0.01 (Analyzed by one‐way ANOVA with post hoc Tukey's multiple comparison test).

### 2.6 In Vivo Cell Infiltration Within TRGHs

The primary objective in engineering the microporous architecture of injectable granular hydrogels is to facilitate tissue regeneration within the targeted region by creating an appropriate microenvironment. For effective endogenous tissue regeneration, the recruitment of cells and the promotion of angiogenesis are critical to initiating the regenerative process [44]. To assess in vivo cell infiltration within our TRGH biomaterials, all granular hydrogel groups were fabricated using microparticles functionalized with RGD peptide and containing vascular endothelial growth factor (VEGF) to promote biological activities such as cell adhesion and infiltration. The L‐GHN hydrogel was injected subcutaneously into mice prior to matrix crosslinking, followed by a short period of transdermal ultraviolet (UV) irradiation to induce matrix crosslinking (Figure 6a). The H‐GH, L‐TRGH, and H‐TRGH hydrogels were injected without subsequent UV irradiation. After seven days, granular hydrogels were retrieved from the injection sites, and the microparticle morphology, infiltrating cells, and vessels were characterized within the L‐GHN, H‐GH, L‐TRGH, and H‐TRGH groups using standard immunohistochemical staining protocols (Figure S9a). Notably, in the L‐TRGH group, microparticle packing remained low even after extrusion; however, some spatial heterogeneity was observed around the outer regions, exhibiting a high degree of cell infiltration and lower interparticle distance compared to inner regions with fewer cells and a higher interparticle distance, suggesting that cells migrated into the hydrogel from surrounding tissues and potentially remodeled the spatial distribution of microparticles (Figure S9b). Quantitative analysis of nuclei coverage area yielded values of ∼0.069, ∼1.85, ∼2.45, and ∼3.15% for the L‐GHN, H‐GH, L‐TRGH, and H‐TRGH groups, respectively, suggesting that TRGHs supported rapid cell invasion from the host tissues. Corresponding CD31+ coverage area measurements were ∼0.069, ∼2.05, ∼5.1, and ∼7.7% for the L‐GHN, H‐GH, L‐TRGH, and H‐TRGH groups, respectively (Figure 6b). These signals represent the invasion of endothelial cells and new blood capillaries, likely in response to the local release of VEGF from the microparticles. The sections were also stained for CD68, a well‐established pan‐macrophage marker [45], to assess the initial immune response following hydrogel implantation (Figure S10). The L‐GHN group demonstrated a significantly reduced CD68‐positive area compared to all other groups, attributable to the lack of cell infiltration associated with the non‐degradable and chemically crosslinked matrix. Conversely, CD68‐positive areas were detected in the H‐GH, L‐TRGH, and H‐TRGH groups, with no significant differences observed among them. These results suggest that macrophage recruitment occurred in the H‐GH and TRGH groups. Furthermore, the presence of the matrix hydrogel within the TRGHs did not affect immune responses relative to the H‐GH group. The H‐TRGH group offers a greater number of cell adhesion moieties due to its high microparticle density, resulting in a more confined interstitial space. In contrast, the L‐TRGH group provides fewer cell adhesion moieties but a more expansive interstitial space. This compensatory effect may account for the absence of significant differences in the areas stained by DAPI and CD31 between the H‐TRGH and L‐TRGH groups. Furthermore, the H‐TRGH group exhibited a greater DAPI‐stained area and a significantly higher expression within the CD31+ area compared to the H‐GH group, indicating a positive effect of the interstitial matrix on cell infiltration at the same packing density. The L‐GHN group exhibited minimal cell infiltration, attributable to its non‐degradable nanoporous matrix hydrogel. In contrast, the H‐GH, L‐TRGH, and H‐TRGH groups demonstrated cell infiltration into the interstitial spaces and adhesion to microparticles (Figure 6c). Furthermore, an increasing trend in cell infiltration was observed, consistent with the in vitro cell spreading assay results (Figure 5).

**FIGURE 6:**
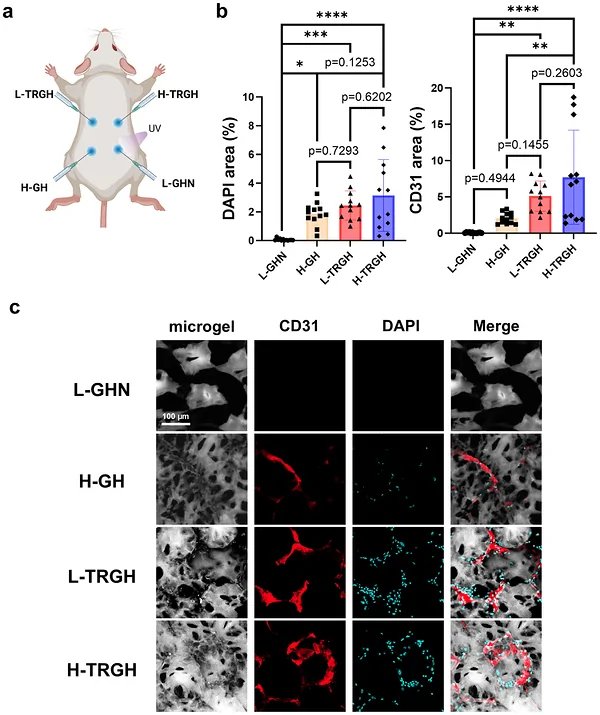
In vivo cell infiltration into granular hydrogels. (a) Schematic illustration depicting the subcutaneous model used to evaluate in vivo cell infiltration. (b) Quantification of DAPI and CD31 expression following seven days of subcutaneous hydrogel injection. (c) Representative confocal images showing microparticles, CD31 fluorescence signal, and nuclei after seven days. ^*^ *p* < 0.05, ^**^ *p* < 0.01, ^***^ < 0.001, ^****^ *p* < 0.0001 (Analyzed by one‐way ANOVA with post hoc Tukey's multiple comparison test).

Taken together, these findings suggest that the matrix hydrogels within TRGHs provide not only temporal support for interparticle spacing but also facilitate cell infiltration. Under non‐injured subcutaneous conditions and at an early time point (7 days), endogenous cell infiltration into TRGHs was greater compared to the H‐GH group. This indicates that the microstructure of granular hydrogels alone can effectively elicit biological responses, and that the interstitial matrix further enhances this effect by stabilizing the microstructure. Future studies should investigate the long‐term effects of varied interparticle distances and particle shapes within TRGHs, as well as the beneficial role of the interstitial matrix, under both non‐injured and injured conditions.

## 3 Conclusions

Here, we develop thermo‐responsive granular hydrogels (TRGHs) with an interstitial matrix that fills the interstitial spaces and undergoes gelation at approximately 20°C, compensating for the lack of mechanical properties observed in granular hydrogels with high interparticle spacing. This system enables the design of loosely packed granular hydrogels without compromising overall mechanical integrity. Variations in the packing density of TRGHs create diverse microenvironments, which in turn influence cell spreading and migration. Furthermore, the interstitial matrix does not impede cell migration and infiltration; rather, it supports these processes when compared to hydrogels of equivalent packing density. Consequently, the TRGH system addresses the design limitations of current granular hydrogel systems by overcoming the trade‐off between mechanical properties and porosity. The TRGH system holds potential for further development as a versatile platform with tailored microarchitectures for tissue regeneration applications.

## 4 Experimental Section

### 4.1 Microparticle Fabrication

Norbornene‐modified hyaluronic acid (NorHA) was initially synthesized through amide coupling between the carboxylic groups of hyaluronic acid (69.4 kDa, Life Biomedical) and 5‐norbornene‐2‐methylamine (Sigma–Aldrich). The resulting NorHA was purified by dialysis against a salt solution for five days, followed by dialysis against deionized water for an additional five days. Subsequently, the NorHA solution was lyophilized, and the degree of modification was determined using proton nuclear magnetic resonance (^1^H NMR) spectroscopy (D_2_O, Bruker AV III 400 HD).

NorHA was dissolved in phosphate‐buffered saline (PBS) to a final concentration of 3 wt.%. Subsequently, lithium phenyl‐2,4,6‐trimethylbenzoylphosphinate (LAP; 1 mg/mL, Sigma–Aldrich) and dithiothreitol (DTT; 4 mm, Bio‐Rad) were added. For fluorescence imaging, either fluorescein isothiocyanate (FITC)‐dextran (2 MDa, 100 µg/mL, Sigma–Aldrich) or cyanine3 (Cy3) tetrazene (10 µg/mL, Lumiprobe) was incorporated into the precursor solution. To promote cell adhesion, 1 mm RGD peptide (GCGYGRGDSPG, Peptide 2.0) was included. For in vivo subcutaneous injection, recombinant human vascular endothelial growth factor (rhVEGF; 1.5 µg/mL, R&D Systems) was added in combination with the RGD peptide.

To fabricate spherical microparticles, a microfluidic device mold was designed featuring two oil inlets positioned on either side of a central polymer inlet, as well as a single outlet, using stereolithography. The mold was cast in polydimethylsiloxane (PDMS; Sylgard 184, Dow Corning) by pouring the polymer onto the plastic mold. After curing at 37°C overnight, the PDMS was peeled off and bonded to a glass slide via oxygen plasma treatment. Subsequently, 1 mm biopsy punches (Fisher) were used to create the inlets and outlet, to which stainless steel 18‐gauge blunt needles (McMaster‐Carr) were attached. Silicone tubing (Accuflow Systems, ABW00001) was then connected to the device. Light mineral oil containing 2% (v/v) Span 80 (Sigma–Aldrich) was flowed at 80 µL/min, while the polymer precursor was introduced at 5 µL/min. Polymer droplets were cured by exposure to UV light (20–30 mW/cm^2^, 2–3 min, Omnicure S1500) within the tubing prior to collection. The collected microparticles were centrifuged to remove excess oil and subsequently washed three times with 1% Tween 20 (Sigma–Aldrich) in PBS.

### 4.2 Fabrication of Granular Hydrogel and Thermo‐Responsive Granular Hydrogel (TRGH)

The fabricated microparticles were packed by centrifugation at 10 000 RCF for 3 min, followed by removal of the supernatant to obtain a highly packed granular hydrogel (H‐GH). To prepare the thermo‐responsive matrix, a hydrogel mixture containing 0.2 g of Pluronic F127 (Sigma–Aldrich) per 1 mL of a PBS/H‐GH solution (7:3, v/v) was stirred at 5°C overnight. Subsequently, the mixture was centrifuged at 5°C with relative centrifugal forces ranging from 100 to 10000 RCF to regulate the packing density, and the excess matrix hydrogel solution was discarded.

### 4.3 Interparticle Space Characterization

Granular hydrogels composed of Cy3‐modified microparticles were fabricated with varying packing densities by applying different centrifugation speeds ranging from 100 to 10000 RCF. These granular hydrogels were subsequently loaded into polydimethylsiloxane (PDMS) molds measuring 6 mm in diameter and 3 mm in height, both with and without the use of a 27‐gauge needle (BD Diagnostic Systems). The microparticles were imaged using an upright Zeiss LSM 800 confocal microscope, acquiring 200 µm z‐stacks across multiple regions of interest. Fluorescence thresholds were then adjusted, and interstitial spacing as well as the aspect ratio of the interstitial spaces were quantified using FIJI software. Additionally, the confocal image files were imported into IMARIS software (version 10.1.1, Oxford Instruments) for three‐dimensional rendering, slice‐through video generation, and quantification of interparticle spacing.

### 4.4 Thermo‐Responsive Mechanical Properties Test

GH, matrix hydrogel composed of 20 wt.% Pluronic F127, and packed TRGHs with low (100 RCF), medium (1000 RCF), and high (10000 RCF) centrifugal forces (denoted as L‐TRGH, M‐TRGH, and H‐TRGH, respectively) were prepared at 5°C. A 20 wt.% Pluronic F127 formulation has been widely used in tissue regeneration applications because it remains flowable at low temperatures and undergoes a sol–gel transition near physiological temperature [28, 46]. Mechanical properties were analyzed using an Anton Paar MCR 702e dynamic mechanical analyzer (DMA) equipped with a 25 mm parallel plate geometry. Hydrogel samples were loaded onto the DMA after setting the plate temperature to 5°C and adjusting the gap to 0.5 mm. Oscillatory shear stress (0.5% strain, 1 Hz frequency) was applied to the hydrogels to monitor storage and loss modulus while the temperature increased from 5°C to 37°C. Subsequently, at 37°C, a strain sweep test was conducted to evaluate shear‐thinning behavior. Additionally, the self‐healing properties of the hydrogels were assessed by monitoring the moduli under alternating shear strains of 0.5% and 100%.

### 4.5 Extrusion Force

The extrusion force of hydrogels was measured using an Arduino‐based force measurement system equipped with a force‐resistive sensor, as previously described [47]. Specifically, when hydrogels were extruded through a 23‐gauge syringe needle, the force‐resistive sensor recorded the analog voltage corresponding to the extrusion force. A calibration curve was established by applying known weights to the sensor and recording the resulting output voltages. Approximately 100 µL of hydrogel was loaded into a syringe fitted with a 23‐gauge needle and extruded at a flow rate of 10 mL/h using a syringe pump. The force sensor was positioned between the syringe plunger and the pump's pusher block to ensure consistent force measurements during extrusion.

### 4.6 3D Printing

A fused deposition modeling (FDM) 3D printer (Ender 3V2, Creality) was modified for extrusion‐based printing following the method established by Domenic et al. [48]. Briefly, the mounting components were fabricated using a Prusa 3D printer with polylactic acid (PLA) filament. The original nozzle part was removed and replaced with the custom‐printed mounting bracket to attach a syringe‐based extrusion system. The modified system was equipped with a 3 mL Luer‐lock syringe coupled to 23 G needles for material extrusion. Material deposition was achieved through mechanical actuation of the syringe piston using the printer's extruder gear mechanism. STL files were converted to G‐code using Cura slicing software (Ultimaker). The ambient temperature was maintained between 20°C and 25°C, while the printing bed temperature was consistently held at 37°C throughout the fabrication process. Printing was performed at speeds ranging from 5 to 10 mm/s with a layer height of 0.2 mm. The extrusion rate of the syringe plunger was adjusted to 10–20 mm/min, depending on the quantity of filament extruded, which was influenced by the mechanical properties of the hydrogels.

Filament, tube, and grid structures were fabricated using L‐GH, H‐GH, and TRGHs and subsequently imaged with a Keyence BZ‐X810 inverted fluorescence microscope. Printability was quantified based on the circularity of the printed grid structures, which was analyzed using FIJI software and calculated according to Equation (1), as established by Ouyang et al. [40]

(1) $$Printability=\frac{\pi }{4}\times \frac{1}{C}=\frac{L^{2}}{16A}$$

where C denotes circularity, L represents the perimeter, and A corresponds to the mean area.

### 4.7 In Vitro Spheroid Culture Within Granular Hydrogels

All microparticles were functionalized with RGD peptide during their fabrication. Low‐packed GH with a non‐degradable matrix was prepared by incorporating H‐GH into a hydrogel solution composed of LAP (1 mg/mL), DTT (4 mm), and NorHA (3 wt.%), followed by centrifugation at 100 RCF. H‐GH, L‐TRGH, and H‐TRGH hydrogels were also fabricated with RGD incorporated microparticles. GFP‐expressing fibroblasts (GFP‐NIH3T3, ATCC) at passages 13–15 were utilized for spheroid formation. Cells were cultured in a CO_2_ incubator at 37°C in Dulbecco's Modified Eagle's Medium (DMEM, Corning) supplemented with 10% fetal bovine serum and 1% penicillin/streptomycin. The growth medium was replaced every other day. Cells were seeded at a density of 1000 cells per well onto microwells fabricated from agarose (Invitrogen) templated with Aggrewell 400 (Stem Cell Technologies) and cultured for one day. Following spheroid collection, the spheroids were mixed with hydrogels by pipetting using a positive displacement pipette (Gilson). The spheroid‐hydrogel mixtures were injected through a 23‐gauge needle (BD Diagnostic Systems) into polydimethylsiloxane (PDMS) molds measuring 8 mm in diameter and 5 mm in height, which were covered on both sides with 40 µm nylon mesh (Fisher). The molds were then immersed in growth medium and cultured in a CO_2_ incubator on a two‐dimensional rocker for three days. Cell nuclei were stained with 4′,6‐diamidino‐2‐phenylindole (DAPI), and cell morphology was imaged using an inverted confocal microscope (Zeiss LSM 880). Maximum projection images were generated, and cell areas were quantified using FIJI software. For the assessment of cell viability, cell spheroids were formed using passage 6‐7 fibroblasts (NIH3T3, ATCC), encapsulated within hydrogels, and cultured for one day as previously described above. Subsequently, live/dead staining was conducted utilizing a live/dead staining kit (Invitrogen). Following this, nuclei were stained with Hoechst 33342 (Invitrogen). Quantitative analysis was performed by measuring the percentage area of live cells using FIJI software.

### 4.8 In Vivo Subcutaneous Injection

C57BL/6 mice aged 14 to 16 weeks were obtained from Envigo for the in vivo study. All experimental procedures were conducted in accordance with the NIH Guide for the Care and Use of Laboratory Animals and were approved by Purdue Institutional Animal Care and Use Committee (PACUC protocol # 202500000090). Mice were anesthetized with isoflurane, and the dorsal area was shaved using depilatory cream. Subsequently, L‐TRGH, H‐TRGH, and H‐GH were subcutaneously injected into the dorsal region using a 23‐gauge syringe. For L‐GHN, the hydrogel was injected subcutaneously in slurry form, followed by UV irradiation to induce matrix crosslinking. After seven days, the mice were euthanized, and the hydrogels were harvested.

### 4.9 Histological Analysis

The collected samples were fixed in 10% neutral buffered formalin (Epredia) for 24 h. Subsequently, the samples were sequentially immersed in 15% and 30% sucrose solutions for 24 h each, followed by embedding in OCT compound (Tissue‐Tek) and cryopreservation. The embedded samples were sectioned at a thickness of 20 µm using a cryostat. For Hematoxylin and Eosin staining, the slides were hydrated in distilled water and processed using an H&E stain kit according to the manufacturer's protocol (Vector Laboratories). Additionally, the 20 µm sections were stained with DAPI (1 µg/mL, 4083S, Cell Signaling Technology) and either a CD31 primary antibody (1:50, 550274, BD Pharmingen) or a CD68 primary antibody (1:100, MCA1957GA, BIO‐RAD). This was followed by fluorescent secondary antibodies, specifically Alexa Fluor 647 goat anti‐rat IgG (A‐21247, Invitrogen) or Alexa Fluor 594 donkey anti‐rat IgG (A‐21209, Invitrogen). The slides were then mounted with ProLong Gold Antifade mounting medium (P36930, Invitrogen). Images were acquired using a Keyence BZ‐X810 inverted fluorescence microscope or a Zeiss LSM 880 inverted confocal microscope and subsequently processed with FIJI software.

### 4.10 Statistics

All graphical and statistical analyses were performed using GraphPad Prism software (version 10.6.1). Differences were assessed using one‐way or two‐way analysis of variance (ANOVA) followed by Tukey's post hoc multiple comparison test, with significance indicated as ^*^(*p* < 0.05), ^**^(*p* < 0.01), ^***^(*p* < 0.001), and ^****^(*p* < 0.0001). For the analysis of interparticle spacing parameters, including Feret diameter, minimum Feret diameter, and aspect ratio, the Kruskal‐Wallis test with Dunn's post hoc multiple comparison test was employed, with significance denoted by the same *p*‐value thresholds.

## Conflicts of Interest

The authors declare no conflicts of interest.

## Supporting information




**Supporting File**: smll75043‐sup‐0001‐SuppMat.docx.

**Supporting Movie**: smll75043‐sup‐0002‐MovieS1.mp4.

## Data Availability

The data that support the findings of this study are openly available in Zenodo at https://doi.org/10.5281/zenodo.21421914.
