# Supplementary material for: Reconciling Granular Hydrogel Microstructure and Mechanics via a Temporary Thermo‐Responsive and Cell‐Invadable Matrix
Source: Small. 2026 Aug 6;22(56):e75043. doi: 10.1002/smll.75043 (PMC13653342; doi:10.1002/smll.75043)
Supplement: Supplementary file 1 [file SMLL-22-e75043-s002.docx]

Supporting Information

**Reconciling Granular Hydrogel Microstructure and Mechanics via a Temporary Thermo-Responsive and Cell-Invadable Matrix**

*Jun Kim, Soyeon Kwon, Taimoor H. Qazi^*^*

Jun Kim, Soyeon Kwon, Taimoor H. Qazi

Weldon School of Biomedical Engineering

Purdue University

West Lafayette 47907, IN, USA

*Corresponding author email: [tqazi@purdue.edu](mailto:tqazi@purdue.edu)

**Keywords**: Microgels, Injectable hydrogels, wound healing, Tissue engineering

Figure S1. ^1^H-NMR spectra of norbornene-modified hyaluronic acid (NorHA). Methyl group peaks and norbornene peaks are indicated in red and blue, respectively.

Figure S2. Interparticle space in granular and thermo-responsive granular hydrogels varies with packing density after extrusion through a 27G needle. ***p < 0.001, ****p < 0.0001 (Analyzed by one-way ANOVA with post hoc Tukey’s multiple comparison test)

Figure S3. Interstitial area morphology analysis of granular and thermo-responsive granular hydrogels varies with packing density a) before and b) after extrusion through a 27G needle. *p < 0.05, **p < 0.01, ***p < 0.001, ****p < 0.0001. The number of interstitial areas was analyzed by one-way ANOVA followed by Tukey’s multiple comparisons test. Feret diameter, minimum Feret diameter, and aspect ratio were analyzed using the Kruskal–Wallis test followed by Dunn’s multiple comparisons test.

Figure S4. Mechanical property recovery of a) GH, b) matrix, and c, d, e) TRGHs under alternating shear strain change at 37 °C. f) Time taken for modulus recovery and g) recovered storage modulus. ***p < 0.001 compared with all other groups (Analyzed by one-way ANOVA with Tukey’s post hoc multiple comparison test)

Figure S5. Extrusion force measurements of L-GH, L-TRGH (at 5°C and 37°C), H-GH, and H-TRGH (at 5°C and 37°C) through a 23 G needle. a) Extrusion force versus time plots. b) Quantitative analysis of the mean extrusion force of the hydrogels. *p < 0.05, ****p < 0.0001 (Analyzed by one-way ANOVA with post hoc Tukey’s multiple comparison test)

Figure S6. Extrusion and 3D printing of matrix hydrogels. a) Extrusion test and 3D-printed filament. b) 3D printing of tubular and grid structures.

Figure S7. 3D printing of tubular structures utilizing a matrix and TRGHs ink on a printing bed maintained at 37 °C.

Figure S8. In vitro cell viability of 3D spheroid culture within L-GHN, H-GH, L-TRGH, and H-TRGH. a) Representative images of live (calcein AM labeling; green) and dead (propidium iodide labeling; red) cells within the spheroids after one day of encapsulation in the hydrogels. b) Quantitative analysis of cell viability based on the proportion of live cells. *p < 0.05 (Analyzed by one-way ANOVA with post hoc Tukey’s multiple comparison test)

Figure S9. Granular hydrogel morphology and cellular infiltration in an in vivo subcutaneous model. a) Hematoxylin and eosin (H&E) staining of implanted hydrogels after 7 days. b) Fluorescence images showing microgels (green), nuclei (blue), and CD31 (red).

Figure S10. Granular hydrogel morphology and macrophage infiltration in an in vivo subcutaneous model. a) Fluorescence images indicating microgel (green), CD68 (red), and nuclei (blue). b) Quantitative data of CD68 positive area fraction. *p < 0.05 (Analyzed by one-way ANOVA with post hoc Tukey’s multiple comparison test)
